# Interns’ perspectives on impacts of the COVID-19 pandemic on the medical school to residency transition

**DOI:** 10.1186/s12909-021-02777-7

**Published:** 2021-06-07

**Authors:** Ariel S. Winn, Matthew D. Weaver, Katherine A. O’Donnell, Jason P. Sullivan, Rebecca Robbins, Christopher P. Landrigan, Laura K. Barger

**Affiliations:** 1 Division of General Pediatrics, Department of Pediatrics, Boston Children’s Hospital, 300 Longwood Avenue, Boston, MA 02115 USA; 2 Department of Pediatrics, Harvard Medical School, Boston, MA USA; 3 Division of Sleep and Circadian Disorders, Departments of Medicine and Neurology, Brigham and Women’s Hospital, Boston, USA; 4 Division of Sleep Medicine, Harvard Medical School, Boston, MA USA

**Keywords:** Medical education, Workforce, UME, GME, COVID-19

## Abstract

**Background:**

The COVID-19 pandemic resulted in disruptions to medical school training and the transition to residency for new post-graduate year 1 resident-physicians (PGY1s). Therefore, the aim of this study was to understand the perspectives of United States PGY1s regarding the impact of the pandemic on these experiences. Our secondary aims were to understand how desire to practice medicine was impacted by the pandemic and whether PGY1s felt that they were able to meaningfully contribute to the COVID-19 response as students.

**Method:**

We conducted a national, cross-sectional study of PGY1s who had recently graduated from medical school in 2020. A survey was distributed to PGY1s from across specialties, in programs distributed throughout the United States. It included questions about medical school training during the pandemic, impact on graduation timing and transition to internship, concerns about caring for patients with COVID-19, desire to practice medicine, and ability to meaningfully contribute to the pandemic. Findings are presented using descriptive statistics and univariate logistic regression models.

**Results:**

1980 PGY1s consented to participate, 1463 completed the survey (74%), and 713 met criteria for this analysis. 77% of PGY1s reported that the pandemic adversely affected their connection with their medical school communities, and 58% reported that the pandemic impeded their preparation for intern year. 4% of PGY1s reported graduating medical school and practicing as an intern earlier than their expected graduation date. While the majority of PGY1s did not have a change in desire to practice medicine, PGY1s with concerns regarding personal health or medical conditions (OR 4.92 [95% CI 3.20–7.55] *p* < 0.0001), the health or medical conditions of others in the home (OR 4.41 [2.87–6.77], *p* < 0.0001]), and PGY1s with children (OR 2.37 [1.23–4.58], *p* < 0.0001) were more likely to report a decreased desire.

**Conclusions:**

The COVID pandemic disrupted the social connectedness and educational experiences of a majority of PGY1 residents in a sample of trainees in United States training programs. Those with health concerns and children had particularly challenging experiences. As the current and subsequent classes of PGY1s affected by COVID-19 proceed in their training, ongoing attention should be focused on their training needs, competencies, and well-being.

**Supplementary Information:**

The online version contains supplementary material available at 10.1186/s12909-021-02777-7.

## Introduction

The Coronavirus disease 2019 (COVID-19) pandemic has caused unprecedented disruptions to healthcare systems and exerted a profound effect on medical education and training [1, 2]. On March 17th, 2020, the Association of American Medical Colleges and Liaison Committee on Medical Education released a joint statement recommending the suspension of medical students’ participation in activities that involve patient contact [3]. Although this policy has since been revised [4], it had immediate and significant ramifications on the clinical experiences of medical students. Additionally, given the need for physical distancing, in-person lectures were either cancelled or transitioned to virtual formats [2]. Although exemplary innovations in medical student education occurred [5, 6], there was also a loss of many traditional learning opportunities that are vital to medical school training and the transition to residency [7].

After medical school, in the United States, new doctors enter residency to further their training in their chosen specialty (e.g., Pediatrics or General Surgery). The first year of residency is often termed internship. Trainees are categorized by their postgraduate year and new doctors in their first year of residency are referred to as post-graduate year 1 resident-physicians (PGY1s). Graduating medical students transitioning to residency and assuming new roles as PGY1s faced unique challenges. Some lost scheduled medical school opportunities designed to better prepare them for their internships, such as critical clinical rotations [8] or internship preparatory experiences [9–11]. Some faced challenging decisions, such as whether or not to graduate early and practice as a doctor prior to their internship start date [12, 13], a unique option that was available to some students in order to assist with the pandemic response. Intern orientations are typically a time when new interns meet their co-interns and receive critical training aimed at easing the transition to internship. In the setting of physical distancing, these orientations were significantly altered and often held virtually. In addition, graduating medical students entered a residency training environment significantly disrupted by the COVID-19 pandemic [14]. Lastly, many trainees had concerns about their personal risk of COVID-19 infection [15], especially at the onset of the pandemic when information about COVID-19 was still evolving.

Despite these disruptions, to our knowledge, there have been no studies to understand the unique experiences of these new doctors. Therefore, the goal of this study was to understand the perspectives of new United States PGY1s regarding the impact of the COVID-19 pandemic on their medical school training and transition to residency. Our secondary aims were to understand how desire to practice medicine was impacted by the pandemic and whether PGY1s felt that they were able to meaningfully contribute to the COVID-19 response as students.

## Methods

We conducted a national, cross-sectional survey of PGY1s at United States residency training programs who graduated from medical school in 2020.

### Survey design

To assess the experiences of PGY1s who had recently graduated and transitioned to internship amidst the COVID-19 pandemic, we designed a voluntary and anonymous survey that was embedded within the ninth year of a longitudinal nationwide study of resident-physician schedules, safety, health and well-being [16]. The study’s baseline survey captured demographic information and information on specialty and residency program. Nineteen questions were added to the current year’s baseline survey (51 questions) to assess how the COVID-19 pandemic affected medical school training and transition to residency (Supplemental Content Survey 1). These questions evaluated impact to medical school training, impact on graduation timing, transition to internship, concerns about taking care of patients with COVID-19, desire to practice medicine and ability to meaningfully contribute to the COVID-19 pandemic. Questions were designed by the study team, comprised of members with collective expertise in clinical research, survey design, medical school education, and residency training. A member of the study team (A.S.W.) performed cognitive interviewing and pilot testing with four graduating medical students to refine the instrument before deployment. This led to the adjustment of wording to several question to improve clarity and the removal of questions that were challenging to answer and deemed less important. We estimate that the total survey took approximately 10 min to complete.

### Study procedures

We followed a multi-pronged recruitment approach with the intent of reaching all graduating medical students who matched to a United States residency program. We contacted medical school deans, program directors, designated institutional officials, and affinity medical groups and requested they forward our email advertisements to their constituents. Potential participants who visited the website were able to read about the study and provide electronic consent. Participants received a $25 gift card for completion of the baseline survey and the four monthly surveys that were part of the parent study. They were also entered into a random drawing for a larger cash prize. On June 28, 2020 individual password-coded links to the baseline survey (which included questions regarding training experiences during COVID, as described above) were emailed to all PGY-1 s who consented to participate. The survey administration methodology has been extensively field tested and refined through its administration in prior years [16–18].

### Statistical analysis

The study population was defined as respondents who graduated medical school in 2020 in order to limit the analysis to those whose training experience was potentially altered by the COVID-19 pandemic. We excluded respondents who had not completed the survey by August 1, 2020 and those who did not answer the question querying the impact of the pandemic on components of their medical training. Descriptive statistics were used to characterize responses from the study sample. The distribution of continuous variables was assessed to determine the most appropriate descriptive presentation. Impact of the COVID-19 pandemic on components of medical school training, change in desire to practice medicine, and satisfaction with ability to respond to the pandemic were collected using 5-point Likert-type scales (Supplemental Content Survey 1). The study team decided to a priori dichotomize these variables in our analysis in order to facilitate interpretation. For example, desire to practice medicine was initially on a 5-point scale (significantly increased, somewhat increased, no change, somewhat decreased, significantly decreased). This was dichotomized to decreased desire (included both significantly and somewhat decreased) and no change/increased (included all other response options). Univariate logistic regression models were used to determine factors associated with concern about caring for patients with COVID-19, satisfaction with ability to respond to the COVID-19 pandemic and change in desire to practice medicine. All statistical analyses were conducted using Stata/SE 15.1 (College Station, TX).

The Human Research Committee of Brigham and Women’s Hospital and Mass General Brigham approved all the study procedures, and all the participants provided electronic written informed consent. All methods were carried out in accordance with relevant guidelines and regulations.

## Results

**Fig. 1:**
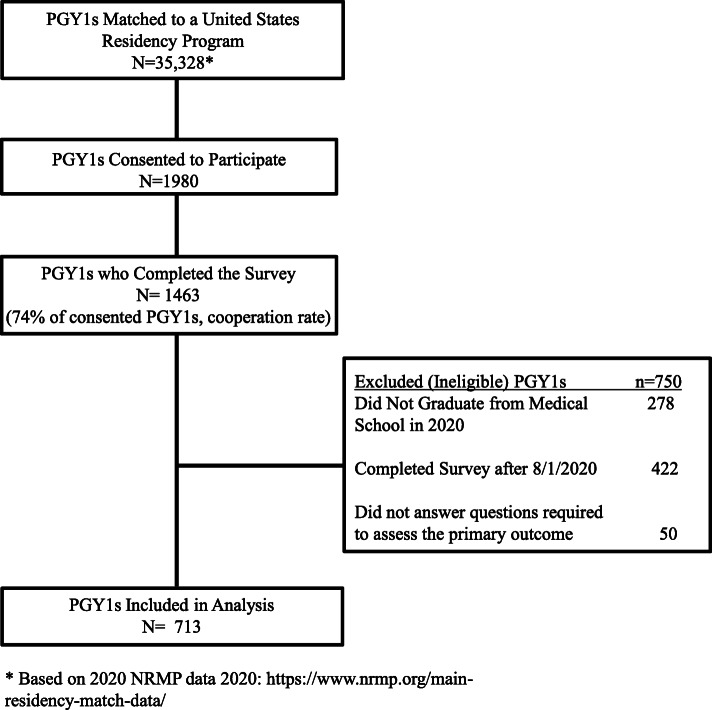
Flow Diagram of Enrollment

The study sample included 713 PGY1s (Fig. 1) from 44 states as well as Puerto Rico with respondent demographics listed in Table 1. Compared to all residents listed in the ACGME Data Resource Book from 2019 to 2020 [19], there was a greater percentage of female residents in our cohort (64% vs 45.1%). The study cohort consisted of 85.0% in medical specialties, 14.3% surgical specialties and 0.7% not reported, compared with 83.7% medical specialties and 16.3% surgical specialties in the National Residency Matching Program cohort [20].

**Table 1:** Demographics of Study Population

	Respondents n (%) *N* = 713
Age (in years, n, mean, SD)	29 (4)
Sex	
Male	256 (36)
Female	453 (64)
Not Reported	4 (1)
Ethnicity	
Hispanic or Latino	27 (4)
Not Hispanic or Latino	680 (95)
Not Reported	6 (1)
Race	
American Indian or Alaskan Native	2 (0)
Asian	160 (22)
Black or African American	21 (3)
Native Hawaiian or other Pacific Islander	1 (0)
White	484 (68)
Other	14 (2)
Multiple	28 (4)
Not Reported	3 (0)
Medical School Location	
United States	678 (95)
International	35 (5)
PGY-1 Year Specialty	
Anesthesia	57 (8)
Emergency Medicine	71 (10)
Family Practice	86 (12)
General Surgery and Surgical Subspecialties	50 (7)
Internal Medicine	153 (21)
Neurology	7 (1)
Obstetrics	47 (7)
Pathology	5 (1)
Pediatrics	99 (14)
Psychiatry	38 (5)
Other including combined training	95 (13)
Not reported	5 (1)
Residency Hospital Setting	
Community	217 (30)
University	494 (69)
Not reported	2 (0)

### Impact to medical school training

**Fig. 2:**
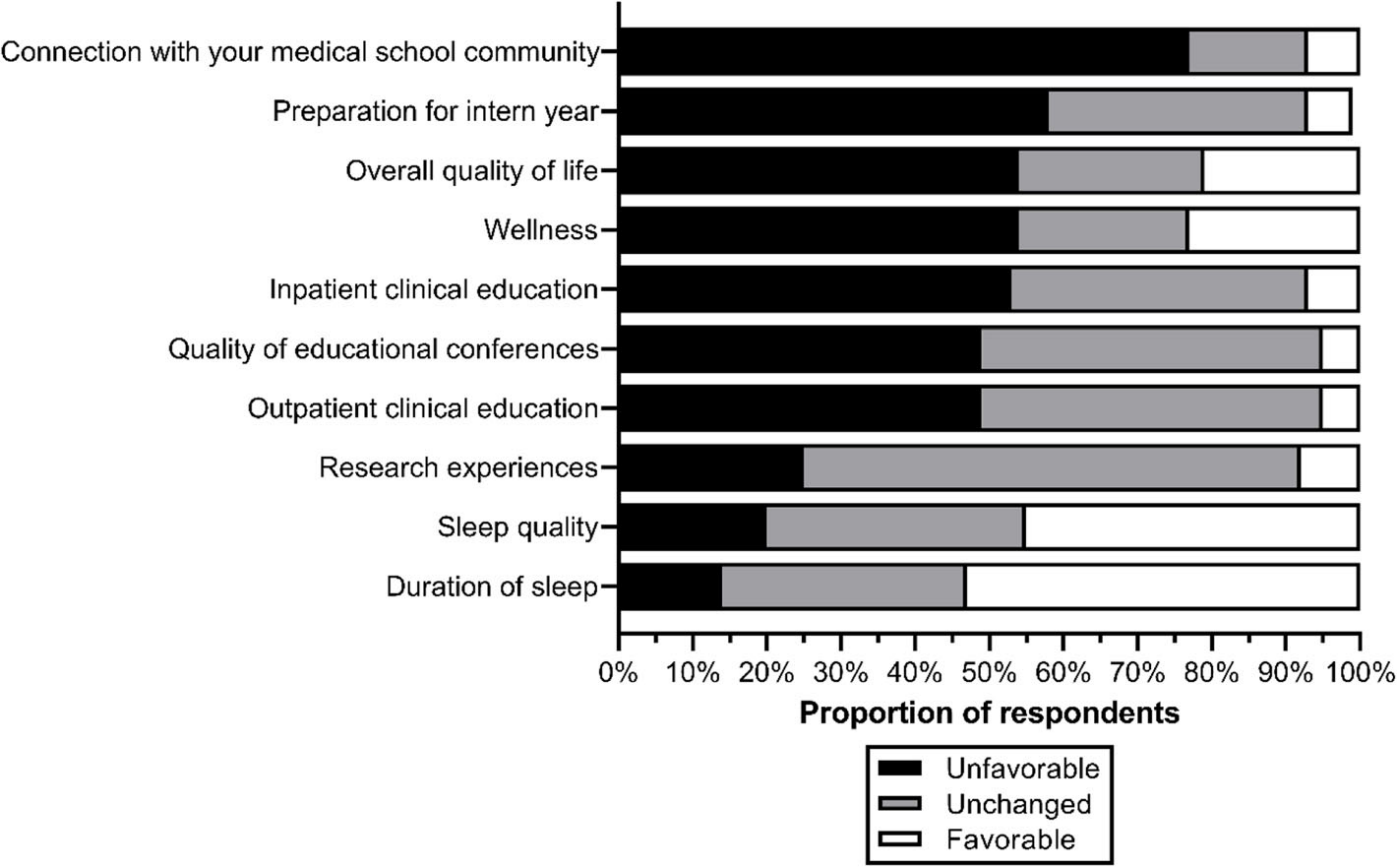
Perceived impact of the COVID-19 pandemic on specific impacts of medical school training

Respondents described a largely unfavorable impact of the COVID-19 pandemic on their medical school training experiences (Fig. 2), except for sleep quality and duration of sleep (53 and 45% reported favorable changes, respectively). The two aspects of medical training most negatively impacted were connection with medical school community (77% unfavorable) and preparation for intern year (58% unfavorable).

**Fig. 3:**
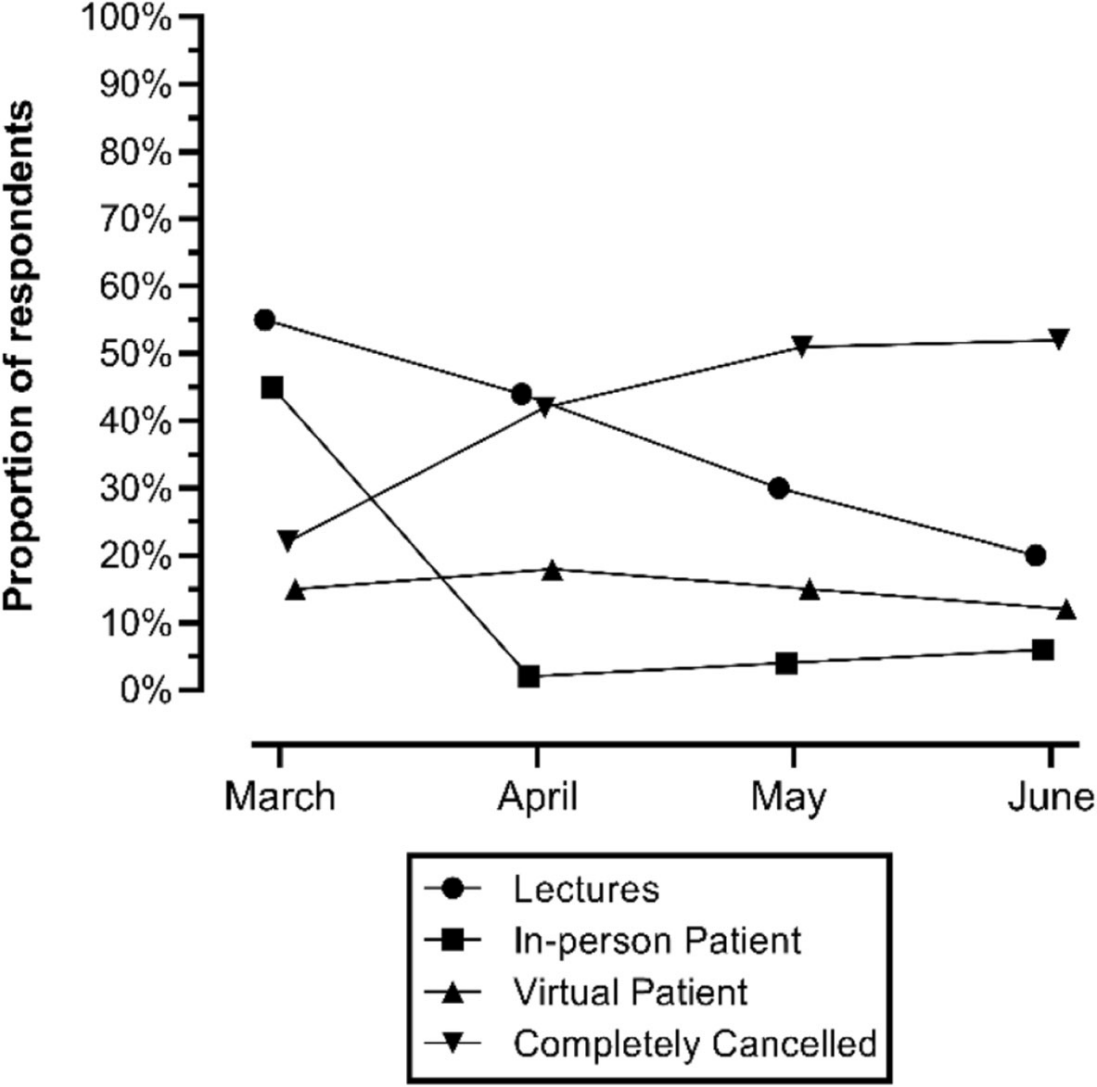
Breakdown of Clinical Experiences by Month for those Scheduled for Clinical Experiences

From March through June, respondents decreasingly reported being scheduled for clinical experiences (March 247/713 residents (34.6%), April 13/713 residents (1.8%), May 11/713 residents (1.5%), June 8/713 residents (1.1%)). The breakdown of what was included in scheduled clinical experiences is displayed in Fig. 3 and indicate that in April through June, as concerns about the pandemic became widespread, 42–52% of clinical experiences were completely cancelled. A minority of respondents who were scheduled for clinical rotations reported in-person (2–6%) or virtual patient experiences (12–18%). There were more varied experiences in March, likely reflecting an adjustment in the clinical experiences of students resulting from evolving national guidance [3].

Only 5.5% (39/713) of respondents reported caring for patients with diagnosed or suspected COVID-19 as a medical student. Of those, 50% reported doing so as part of their medical school curriculum and 50% as part of a voluntary experience.

### Impact on graduation timing

21% (151/711) of PGY1s reported graduating medical school earlier than their expected graduation date. However, of those, only 21% (31/151), which reflects 4% of the entire study population, reported receiving a license and practicing as an intern before their expected residency start date. Of the 31 respondents that reported graduating early and practicing as an intern before their expected residency start date, 77% practiced only at a site affiliated with their medical school, 7% practiced only at a site affiliated with their future residency program, and an additional 7% practiced both at a site affiliated with their medical school as well as a site affiliated with their future residency program.

### Transition to internship

The majority of respondents reported a combination of virtual and in-person activities during their PGY1 orientation (7% completely virtual, 66% mostly virtual with a few in-person activities, 22% mostly in-person with a few virtual activities, and 6% completely in-person). Despite the logistical challenges, 86% (613/712) reported being able to be certified in all required life support training (i.e., ACLS, BLS, PALS). 77% of respondents reported receiving formal training/education in Personal Protective Equipment (PPE). Despite the training, at the time of survey completion (during the first month of residency), only 37% (264/713) reported feeling very or extremely competent in its use.

### Residents with concerns about taking Care of Patients with COVID-19

Concerns about taking care of patients with COVID-19 secondary to either a personal health or medical condition (26%, 182/710) or that of someone living in their home (32%, 226/709) were fairly common. These respondents reported a variety of schedule modifications listed in Table 2. Those that chose “other” listed a variety of schedule modifications. An example includes moving higher risk rotations to later in the year.

**Table 2:** Breakdown of Schedule Modifications for Residents with concerns about taking care of COVID-19 patients secondary to either a personal health or medical condition or that of someone living in their home

	Personal Health n (%) *N* = 182	Health of Someone Living in Home n (%) *N* = 226
Not modified based on resident preference	97 (53)	114 (50)
Would have liked it to be modified but residency program did not allow special accommodations	17 (9)	17 (8)
Remain on normally scheduled rotations but do not take care of patients with diagnosed with COVID-19	43 (24)	62 (27)
Placed on rotations that are considered less high risk	10 (5)	16 (7)
Engaged in telehealth but not direct patient care	3 (2)	2 (1)
Decided to take a leave of absence	0 (0)	0 (0)
Required to take a leave of absence	0 (0)	0 (0)
Other	12 (7)	15 (7)

### Other impacts

The majority of respondents did not have a change in desire to practice medicine as a result of the pandemic (67%, 475/710) with a relatively equal number of residents reporting an increased desire (17%, 123/710) and a decreased desire (16%, 112/710). Residents with concerns regarding personal health or medical conditions (OR 4.92 [95% CI 3.20–7.55] *p* < 0.0001) or the health or medical conditions of others in the home (OR 4.41 [95% CI 2.87–6.77), *p* < 0.0001]) were more likely to report a decreased desire to practice medicine. This was also true for residents with children (OR 2.37 [95% CI 1.23–4.58], *p* < 0.0001). Resident-physicians specializing in internal medicine did not have a decreased desire to practice medicine as compared to other specialties (95% CI OR 0.90 [0.54–1.50], *p* = 0.70). No other demographic factors listed in Table 1 were associated with a decreased desire to practice medicine.

More than half of all respondents reported being not at all or only somewhat satisfied (59%,420/709) with their ability to meaningfully contribute to the COVID-19 pandemic. Respondents who cared for COVID-19 patients (*n* = 39) as part of their medical school experience were no more likely to report satisfaction with their ability to contribute (OR 1.49; 95% [CI 0.77–2.86], *p* = 0.24). However, respondents who graduated and practiced as an intern early (*n* = 31) were more likely to report satisfaction with their contribution (OR 2.76 [1.30–5.86], *p* = 0.008).

## Discussion

The transition from medical school to internship is a critical milestone for physicians. We found that most activities surrounding this transition were altered by the COVID-19 pandemic, including disruptions to clinical activities, graduation, and onboarding. Additionally, we found that PGY1s perceived the COVID-19 pandemic to have an unfavorable impact on most aspects of medical school training and that many were not fully satisfied with their ability to meaningfully contribute to the pandemic. Governing bodies [3] and medical schools were forced to rapidly adapt to the constraints placed on medical education by the pandemic. Therefore, it is unsurprising that recently graduated PGY1s generally perceived the impact on their medical school training to be unfavorable. The exception relates to sleep quality and duration of sleep, which is not fully explored in this study, but may relate to cancelled clinical and social activities allowing for more time for sleep.

Self-determination theory postulates that the three basic needs of autonomy, competence and relatedness are necessary to stimulate and sustain intrinsic motivation [21]. Our study indicates that the COVID-19 pandemic had the most unfavorable impact on PGY1s’ sense of connection with their medical school community and preparation for intern year, which directly bear on relatedness and competence. It is critical that we better consider how to support connection amongst medical school and residency communities, through both in-person and virtual activities [22]. This is particularly important during this critical transition as many trainees are entering a residency community knowing very few other co-residents, at a time when in-person gatherings during intern orientation are limited. Fortunately, innovations developed by medical schools and residency programs to foster relatedness and community during the pandemic will almost certainly continue to benefit new physicians after the pandemic. Our findings about preparedness are consistent with those recently published by Choi et al., who found that final year medical students in the United Kingdom (UK) also felt less prepared for Foundation Year 1, the equivalent of internship [8]. Interestingly, a study by Patel et al., found that in addition to a desire to contribute, a motivating factor for UK medical students to volunteer clinically early on in the pandemic, was to develop skills and gain experience thereby likely leading to better preparedness [23]. Fortunately, medical schools are innovating rapidly and creatively to meet the needs of their students and better prepare them for their internship, including integrating students back into sub-internships, integrating students into telehealth activities [24], offering virtual preparatory experiences [25], and developing virtual objective structured clinical examinations [26]. However, residency programs may also need to provide additional training and support for new residents whose medical school experiences may result in them either being less prepared or perceiving themselves to be less prepared for internship, especially as they enter a residency landscape that is significantly different in the face of the pandemic.

In order to ensure the safety of health care professionals and residents during the pandemic, it is critical they are trained and competent in the use of PPE [27]. Our study cohort reported varying levels of perceived competence, even after orientation and after they had started their first rotations. Future studies are needed to understand the objective competence of resident-physicians; however, our study suggests the need to supplement existing training and develop methods for trainees to demonstrate competence prior to interacting with patients with diagnosed or suspected COVID-19.

Although it is reassuring that the majority of PGY1s did not have a change in their desire to practice medicine, certain populations were differentially affected. Amidst school and daycare closures, parents across the country have been forced to negotiate between their roles at work and at home. This is a particular challenge for resident-physicians who often work irregular schedules and up to 80 h per week, with limited flexibility to work from home [28]. Thus, it is unsurprising that these resident-physician parents have a decreased desire to practice medicine as compared to their peers. As the challenges around parenting during the pandemic continue, medical schools, residency programs and institutions should consider implementing supports for these parents, including but not limited to childcare support. Additionally, PGY1s who reported concerns about taking care of patients with COVID-19 secondary to either a personal health or medical condition or that of someone living in their home also reported a decreased desire to practice medicine as compared to their peers. This study also describes some of the potential accommodations offered to these trainees which could be used to guide national policies or guidance on how to best accommodate these trainees so that program directors do not have to navigate these conversations on their own.

Given that our study cohort included only a fraction of all PGY1s in the United States, a potential limitation is nonresponse bias. However, we are reassured that the demographic characteristics of our participants are comparable to national demographic characteristics. It is also possible that respondents to this survey were not representative of PGY1s nationally in ways that we did not assess. The advertised topic of the study was schedules, safety, and well-being. Because this secondary topic of COVID-19 medical school experience and transition to residency was not mentioned on the study website, it likely did not attract those with heightened interest in this topic, further mitigating the risk of nonresponse bias. Additionally, for some questions, PGY1s were asked to recall events from several months earlier, potentially resulting in recall bias. However, we attempted to minimize this bias by excluding participants that completed the survey after August 1st. Lastly, the experiences of PGY1s in the United States may be different than the experience of PGY1s in other countries worldwide which may impact generalizability. Nevertheless, the geographically diverse sample and the themes represented in this analysis have important implications for medical school training and transition to residency as we continue to face uncertainty about the future amidst the COVID-19 pandemic and in anticipation of future pandemics.

Future studies should focus on continuing to understand the experiences of medical students and new residents as the pandemic and the response to the pandemic continues to evolve. Additional studies should focus on whether objective competence of graduating medical students has been impacted by the pandemic.

## Conclusion

This study identifies the multiple ways in which the transition to internship among PGY1s was impacted by the COVID-19 pandemic. As the pandemic continues, it is prudent to monitor the experience of resident-physicians and to continue to identify opportunities to improve the experience for future medical students and new resident-physicians. These lessons could also inform responses to future pandemics.

## Supplementary Information

**Additional file 1:.** Supplemental content: survey questions

## Data Availability

The datasets generated and/or analyzed during the current study are not publicly available due to ongoing data collection but will be available from the corresponding author on reasonable request.

## Abbreviations

COVID-19: Coronavirus disease 2019
PGY-1: Post-graduate year 1 resident-physician
