# Supplementary material for: Interns’ perspectives on impacts of the COVID-19 pandemic on the medical school to residency transition
Source: BMC Med Educ. 2021 Jun 7;21:330. doi: 10.1186/s12909-021-02777-7 (PMC8184261; doi:10.1186/s12909-021-02777-7)
Supplement: Supplementary file 1 — Additional file 1:. Supplemental content: survey questions [file 12909_2021_2777_MOESM1_ESM.docx]

# Supplemental Content: Survey Questions

# Demographics

D1. **What is the zip code of your residency teaching hospital?** _______

D2. **What is your current marital status?**

⭘ Married

⭘ Separated

⭘ Divorced

⭘ Widow/widower

⭘ Never married

D3. **Who lives in the same household as you?**

⭘ Child/children

⭘ Spouse/Partner

⭘ Other family members

⭘ Roommate

⭘ Pet

D4. **How many children live with you?**

|  |  |
| --- | --- |

| Hispanic or Latino: A person of Cuban, Mexican, Puerto Rican, South or Central American, or other Spanish culture or origin regardless of race |
| --- |

D5. **What is your ethnicity? (***Choose one***)**

⭘ Hispanic or Latino

⭘ Not Hispanic or Latino

D6. **What is your race? (***Please select all that apply***)**

⭘ American Indian or Alaskan Native

⭘ Asian

⭘ Black or African American

⭘ Native Hawaiian or other Pacific Islander

⭘ White

⭘ Other

| **Please use the categories that most closely reflect your recognition in the community for purposes of reporting mixed racial and/or ethnic origins.**  **American Indian or Alaskan Native**: A person having origins in any of the original peoples of North, Central, or South America, and maintains tribal affiliations or community attachment.  **Asian**: A person having origins in any of the original peoples of the Far East, Southeast Asia, or the Indian subcontinent including, for example, Cambodia, China, India, Japan, Korea, Malaysia, Pakistan, the Philippine Islands, Thailand, and Vietnam.  **Black or African American**: A person having origins in any of the black racial groups of Africa.  **Native Hawaiian or Pacific Islander**: A person having origins in any of the original peoples of Hawaii, Guam, Samoa, or other Pacific Islands.  **White**: A person having origins in any of the original peoples of Europe, North Africa, or the Middle East. |
| --- |

# Program

R1 a. **Which of the following best describes your specialty program for this upcoming year?**

⭘ Transitional Internship

⭘ Medical Preliminary Internship

⭘ Surgical Preliminary Internship

R1 B. **Type:**

⭘ Anesthesiology

⭘ Obstetrics/Gynecology

⭘ Dermatology

⭘ Orthopedic Surgery

⭘ Radiation Oncology

⭘ Emergency Medicine

⭘ Ophthalmology

⭘ Radiology

⭘ Family Practice

⭘ Otolaryngology

⭘ Surgery-General

⭘ Internal Medicine

⭘ Pediatrics

⭘ Internal Medicine/Pediatrics

⭘ Neurological Surgery

⭘ Pathology

⭘ Urology

⭘ Neurology

⭘ Physical Medicine/Rehab

⭘ Psychiatry

⭘ Other (Not Listed Above). Please Describe: _________________________________

R2. **Would you say your program is predominately community or university based?**

⭘ Community

⭘ University

**Changes to your Medical School Experience:**

The following questions are about your medical school and orientation experiences during the COVID-19 pandemic. When we ask you about your experiences, please consider experiences from March 1, 2020 to the present day. When we ask you about the academic year, please assume that it ends in June, 2020.

C1. Please indicate what was included in your clinical experiences in the final months of medical school? (check all that apply)

|  | Lectures | In-person patient interactions | Virtual patient interactions | Completely cancelled | Not applicable (not scheduled for clinical experiences this month or graduated early) |
| --- | --- | --- | --- | --- | --- |
| March |  |  |  |  |  |
| April |  |  |  |  |  |
| May |  |  |  |  |  |
| June |  |  |  |  |  |

C2. **As a medical student**, did you care for patients with diagnosed or suspected COVID-19? (check all that apply)

⭘ yes, as part of my medical school curriculum

⭘ yes, as part of a voluntary experience

⭘ no

C2a. (if yes to either), please estimate the total number of patients with diagnosed or suspected COVID-19 you have taken care as a medical student? ______

C3. Did you graduate from medical school earlier than your expected graduation date?

⭘ Yes

⭘ No

C3a. (If yes), did you receive a license and practice as an intern before your expected residency start date?

⭘ Yes

⭘ No

C3a1. (If yes), please describe the setting in which you practiced (check all that apply)?

⭘ A healthcare setting affiliated with my medical school

⭘ A healthcare setting affiliated with my future residency program

⭘ A healthcare setting that is neither affiliated with my medical school or future residency program

⭘ Other _____

C3a2. (if yes to license and practice), did you take care of any patients with diagnosed or suspected COVID-19?

⭘ yes

⭘ no

If yes, please estimate the total number of patients with diagnosed or suspected COVID-19 you have taken care of in this setting? ______

C4. How prepared do you feel to start intern (PGY-1) year?

⭘ not at all

⭘ slightly

⭘ moderately

⭘ very

⭘ extremely

C5. Please indicate the impact the COVID-19 Pandemic has had on the following areas of your medical school training:

| Area | Very Unfavorable | Unfavorable | Unchanged | Favorable | Very Favorable |
| --- | --- | --- | --- | --- | --- |
| Inpatient clinical education |  |  |  |  |  |
| Outpatient clinical education |  |  |  |  |  |
| Preparation for intern year |  |  |  |  |  |
| Research experiences |  |  |  |  |  |
| Quality of educational conferences |  |  |  |  |  |
| Wellness |  |  |  |  |  |
| Sleep Quality |  |  |  |  |  |
| Duration of Sleep |  |  |  |  |  |
| Connection with your Medical School Community |  |  |  |  |  |
| Overall Quality of Life |  |  |  |  |  |

C6. How many months has it been since your last clinical experience with direct patient contact? _____

**Orientation and PPE Training:**

O1. Please describe the format of your intern orientation:

⭘ completely virtual

⭘ mostly virtual with a few in person activities

⭘ mostly in person with a few virtual activities

⭘ completely in person

O2. Were you able to be certified in all of your required life support training (e.g. ACLS, BLS, PALS)

⭘ Yes, completely

⭘ Partially

⭘ No, not at all

O3. As part of your residency onboarding, did you receive formal training/education in Personal Protective Equipment (PPE):

⭘ Yes

⭘ No

O4. How competent do you feel in the use of PPE:

⭘ not at all competent

⭘ somewhat competent

⭘ competent

⭘ very competent

⭘ extremely competent

**Other COVID-19 effects:**

O5. How has the COVID-19 pandemic affected your desire to practice medicine?

⭘ Significantly increased desire

⭘ Somewhat increased desire

⭘ No change in desire

⭘ Somewhat decreased desire

⭘ Significantly decreased desire

O6. Did you feel satisfied with your ability to meaningfully contribute to the response to the COVID-19 pandemic?

⭘ not at all satisfied

⭘ somewhat satisfied

⭘ satisfied

⭘ very satisfied

⭘ extremely satisfied

**Special Populations:**

O7. Based on your health or personal medical conditions, do you have concerns about taking care of COVID-19 patients?

⭘ yes

⭘ no

⭘ prefer not to answer

O8. Based on the health or personal medical conditions of someone living in your home, do you have concerns about taking care of COVID-19 patients?

⭘ yes

⭘ no

⭘ prefer not to answer

O9. If yes to either of above, please indicate how your schedule has been modified (please choose the best option)?

⭘ It has not been modified based on my preference

⭘ I would have liked my schedule to have been modified but my residency program did not allow special accommodations

⭘ I remain on normally scheduled rotations but will not take care of patients diagnosed with COVID-19

⭘ I have been placed on rotations that are considered less high risk

⭘ I am not participating in direct patient contact but can engage in telehealth

⭘ I decided to take a leave of absence

⭘ I was required to take a leave of absence

⭘ Other_____________________________________
